# The Divergent Immunomodulatory Effects of Short Chain Fatty Acids and Medium Chain Fatty Acids

**DOI:** 10.3390/ijms22126453

**Published:** 2021-06-16

**Authors:** Qi Hui Sam, Hua Ling, Wen Shan Yew, Zhaohong Tan, Sharada Ravikumar, Matthew Wook Chang, Louis Yi Ann Chai

**Affiliations:** 1 Division of Infectious Diseases, University Medicine Cluster, National University Health System, NUHS Tower Block, 1E Kent Ridge Road, Singapore 119228, Singapore; bchsqh@nus.edu.sg (Q.H.S.); zhaohong_tan@nuhs.edu.sg (Z.T.); sharada_ravikumar@nuhs.edu.sg (S.R.); 2 Department of Biochemistry, Yong Loo Lin School of Medicine, National University of Singapore, Singapore 117597, Singapore; bchlingh@nus.edu.sg (H.L.); wenshanyew@nus.edu.sg (W.S.Y.); bchcmw@nus.edu.sg (M.W.C.); 3 Synthetic Biology for Clinical and Technological Innovation (SynCTI), National University of Singapore, Singapore 117456, Singapore; 4 Synthetic Biology Translational Research Programme, Yong Loo Lin School of Medicine, National University of Singapore, Singapore 117456, Singapore; 5 Department of Medicine, Yong Loo Lin School of Medicine, National University of Singapore, Singapore 119228, Singapore

**Keywords:** microbiome, short chain fatty acids, medium chain fatty acids, cytokine, inflammation, immunity

## Abstract

Fatty acids are derived from diet and fermentative processes by the intestinal flora. Two to five carbon chain fatty acids, termed short chain fatty acids (SCFA) are increasingly recognized to play a role in intestinal homeostasis. However, the characteristics of slightly longer 6 to 10 carbon, medium chain fatty acids (MCFA), derived primarily from diet, are less understood. Here, we demonstrated that SCFA and MCFA have divergent immunomodulatory propensities. SCFA down-attenuated host pro-inflammatory IL-1β, IL-6, and TNFα response predominantly through the TLR4 pathway, whereas MCFA augmented inflammation through TLR2. Butyric (C4) and decanoic (C10) acid displayed most potent modulatory effects within the SCFA and MCFA, respectively. Reduction in TRAF3, IRF3 and TRAF6 expression were observed with butyric acid. Decanoic acid induced up-regulation of GPR84 and PPARγ and altered HIF-1α/HIF-2α ratio. These variant immune characteristics of the fatty acids which differ by just several carbon atoms may be attributable to their origins, with SCFA being primarily endogenous and playing a physiological role, and MCFA exogenously from the diet.

## 1. Introduction

The gut microbiome constitutes a complex pool of metabolites that play a pivotal role in the regulation of diverse bodily functions, from immunity to behavior [1]. Fatty acids make up a significant proportion of these gut metabolites. Our ability to study the gut microbiome in recent years has reignited interest in studying diet–gut–immunity interaction. These metabolites of lipid metabolism can influence the effector arms of the innate and acquired immune system, the details of which are not fully understood. 

Short chain fatty acids (SCFA) have been better studied than medium chain fatty acids (MCFA). SCFA consist of free fatty acids with two to five carbons, the major SCFA produced by gut bacteria are acetic (ethanoic) acid (two carbons), propanoic acid (three carbons), and butyric acid (four carbons). These SCFA are the principal SCFA focused on in most studies, including our present study, and are present in the gut in approximate molar ratios of 60:20:20 [2,3]. In our study, we also include lactic acid as it is also produced by the gut bacteria, though quickly metabolized to form butyric acid or propanoic acid [2]. Valeric acid (also known as pentanoic acid), isovaleric acid and 2-methylbutanoic acid, all consisting of five carbons, are also produced by gut bacteria but in much smaller amounts, and their roles in the human body have been much less well-characterized [3]. All these SCFA are produced as by-products of fermentation of fiber and resistant starches by bacteria, mainly of the phyla *Bacteroidetes* and *Firmicutes* in the gut [4]. 

The MCFA are slightly longer than SCFA, containing 6 to 10 carbon atoms, from hexanoic acid (6 carbons), also known as caproic acid, octanoic acid (8 carbons), also known as caprylic acid, and decanoic acid (10 carbons), also known as capric acid. MCFA are found in the diet in the form of triacylglycerol and metabolized in the body as energy sources, such as in palm kernel (7%) and coconut oil (14%) [5,6]. These MCFA are used in the body as an energy supply for high intensity exercise as they oxidize rapidly [7]. Brain cells, such as astrocytes, can further metabolize MCFA to ketone bodies to be used as energy for the brain [8]. As such, MCFA have been the subject of much research in the context of energy metabolism and nutrition. In nutrition literature, MCFA have generally been compared with long chain fatty acids (LCFA), and MCFA have generally been considered less- or even anti-inflammatory compared to LCFA. When the LCFA oleic acid was compared to the MCFA octanoic, and decanoic acid, it showed that in human liver cells, TNFα, IL-1β, IL-6, and IL-8 were significantly upregulated by oleic acid compared to octanoic and decanoic acid [9]. In another study, octanoic acid enhanced inflammatory IL-8 secretion by 35 to 40% in a cell line of human fetal intestinal epithelial cells, while oleic acid gave a larger increase of 110 to 140% [6].

The SCFA have been reported to have anti-inflammatory propensity [10]. This has been linked to reduced NF-κB activity and increased prostaglandin E2 release [11,12,13]. To date, most research into MCFA though has been focused on the macro-effects on humans, such as diet and obesity. There are limited studies investigating their immunomodulatory properties, and not many studies comparing the MCFA with the SCFA in terms of their properties on the immune system. 

In this article, we aim to compare and explore the immunomodulatory properties of the individual 2- to 4-carbon moieties of SCFA and 6- to 10-carbon moieties of MCFA. 

## 2. Results

### 2.1. SCFA Down-Modulate the TLR4 Pathway

We investigated the inflammatory pathways triggered by the SCFA and MCFA by stimulating human peripheral blood mononuclear cells (PBMC) over an increasing concentration of SCFA or MCFA (2, 20 and 200 µM, derived from concentrations reported in the literature [14,15]). PBMC stimulated with SCFA or MCFA alone did not show any observable increase in cytokine levels, of all the cytokines we tested, compared to the control PBMC cells. 

**Figure 1:**
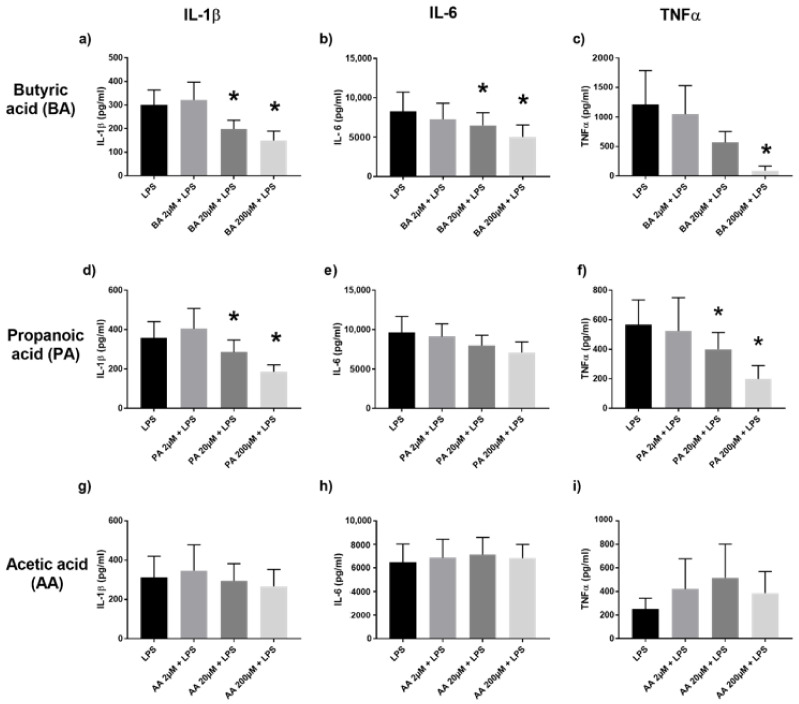
Cytokine production of IL-1β, IL-6 and TNFα in the supernatants of PBMC stimulated with LPS and 2, 20, 200 µM of SCFA, assessed by Enzyme-Linked Immunosorbent Assay (ELISA). Human PBMC were co-incubated with LPS and butyric acid (BA) (**a**–**c**), propanoic acid (PA) (**d**–**f**), or acetic acid (AA) (**g**–**i**). Values are presented as mean ± standard error of the mean (SEM), results from at least 7 subjects were used. Wilcoxon signed rank test to determine significance for pair-wise comparisons with the LPS control, * *p* < 0.05 is the cytokine level difference compared to the control LPS group (black bar).

We stimulated human PBMC with SCFA or MCFA, as mentioned above, in the presence of *Escherichia coli* lipopolysaccharide (LPS), a TLR4 ligand, Pam3Cys-Ser-(Lys)4 (P3C), a TLR2/TLR1 agonist, or heat-killed *Candida albicans* (CA), a gut commensal and complex ligand that is a potent inflammatory stimulator of the PBMC [16,17,18]. In PBMC co-stimulated with LPS and the respective SCFA, we observed that the 4-carbon butyric acid had the most immunomodulatory potency over 3-carbon propanoic acid, 3-carbon lactic acid, and 2-carbon acetic acid, which was the least potent. Butyric acid induced a dose-dependent attenuation of interleukin-1 beta (IL-1β), interleukin-6 (IL-6), and tumor necrosis factor-alpha (TNFα) production via TLR4 (Figure 1a–c). The modulatory influence of propanoic acid was also seen on IL-1β and TNFα production (Figure 1d–f). Acetic acid (Figure 1g–i) and lactic acid (Supplementary Figure S1) did not have a marked effect on proinflammatory cytokine production.

Notably, PBMC co-stimulated with P3C exhibited less modulation of cytokine production by SCFA, indicating the limited influence of SCFA on the TLR2 pathway (Supplementary Figure S2). 

### 2.2. Conversely, MCFA Augment the TLR2 Pathway

**Figure 2:**
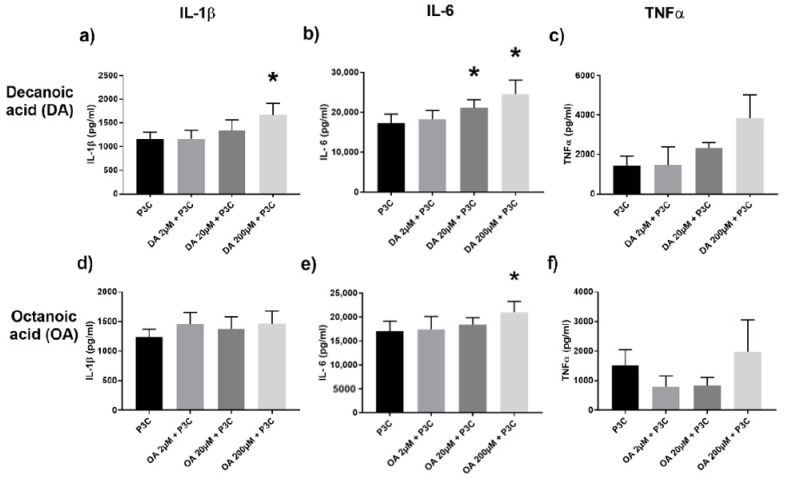
Cytokine IL-1β, IL-6, and TNFα production in the supernatants of PBMC stimulated with P3C and 2, 20, 200 µM of decanoic acid (DA) (**a**–**c**), and octanoic acid (OA) (**d**–**f**). Values are presented as mean ± SEM, results from at least 7 subjects were used. Wilcoxon signed rank test was used to determine significance for pair-wise comparisons with the control, * *p* < 0.05 is the cytokine level difference compared to the control P3C group (black bar).

We stimulated human PBMC with MCFA, which resulted in a dose-dependent, increased cytokine production, particularly IL-1β and IL-6, when PBMC were co-stimulated with P3C. This was most evident with 10-carbon decanoic acid (Figure 2a–c) compared to 8-carbon octanoic acid (Figure 2d–f) and 6-carbon hexanoic acid (Supplementary Figure S3). These results were in contrast with the limited effect exerted by MCFA in the presence of LPS (Supplementary Figure S4), and this pointed to the divergent effect of SCFA and MCFA on the TLR4 and TLR2 pathway, respectively. 

### 2.3. Butyric Acid and Decanoic Acid Induce Variant Responses to Candida

**Figure 3:**
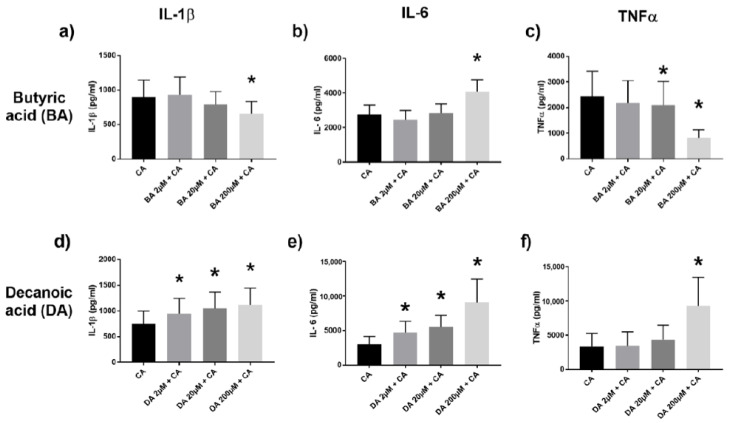
Proinflammatory cytokine IL-1β, IL-6, and TNFα production in the supernatants of PBMC co-stimulated with *Candida albicans* (CA) and various dilutions of butyric acid (BA) (**a**–**c**), or decanoic acid (DA) (**d**–**f**), assessed by ELISA. Values are presented as mean ± SEM, results from at least 7 subjects were used. Wilcoxon signed rank test was used to determine significance for pair-wise comparisons with the control CA group, * *p* < 0.05 is the cytokine level difference compared to the control CA group (black bar).

The yeast *Candida albicans* is a gut commensal and signals through both TLR2 and TLR4 [19,20]. In the presence of CA as stimuli, butyric acid selectively diminished TNFα and IL-1β production while increasing IL-6 levels (Figure 3a–c). However, decanoic acid in the presence of CA augmented IL-1β, IL-6 and TNFα levels (Figure 3d–f). Similar trends were also seen with propanoic acid and octanoic acid, respectively (Supplementary Figure S5).

### 2.4. Anti-Inflammatory IL-10 Increased with SCFA and Decreased with MCFA

The trends of the anti-inflammatory cytokine interleukin-10 (IL-10) were as consistent as anticipated and the inverse of the inflammatory cytokines seen with SCFA and MCFA. We used butyric acid as a representative for the SCFA as it had the most pronounced anti-inflammatory trend. Similarly, decanoic acid, having the most inflammatory response, was used to represent the MCFA.

**Figure 4:**
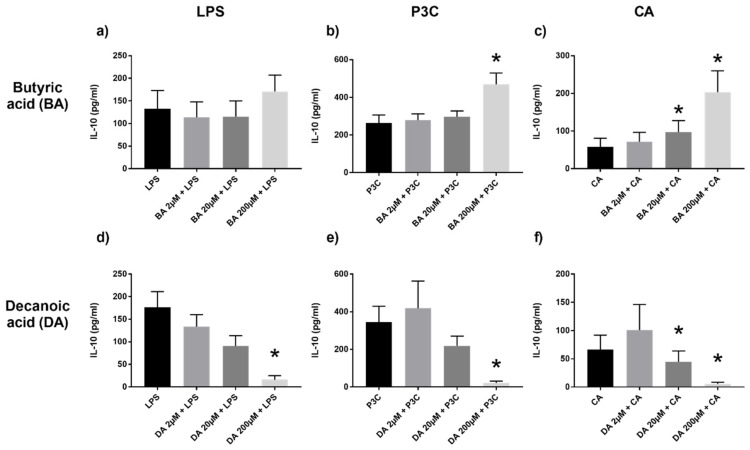
IL-10 production in PBMC stimulated with various dilutions of butyric (BA) (**a**–**c**) or decanoic acid (DA) (**d**–**f**) and LPS, P3C, or CA, assessed by ELISA. Values are presented as mean ± SEM, results from at least 7 subjects were used. Wilcoxon signed rank test to determine significance for pair-wise comparisons with the control group, * *p* < 0.05 is the cytokine level difference compared to the control LPS/P3C/CA group (black bar).

In line with the inflammatory propensity being down-modulated by SCFA, we observed increased IL-10 production with butyric acid; this trend, though, was most evident through P3C and CA (Figure 4a–c). Conversely, IL-10 markedly decreased in a dose dependent manner with decanoic acid (Figure 4d–f), consistent with their pro-inflammatory disposition.

### 2.5. Down Regulation of TRAF3, IRF3, and TRAF6 with SCFA

To further investigate the respective immunomodulatory effects induced by SCFA and MCFA, we studied candidate signaling moieties by studying their transcription profiles. Tumour necrosis factor receptor associated factor (TRAF)3, TRAF6 and interferon regulatory factor (IRF)3 mediate downstream signaling following ligation of TLR2 and TLR4. 

**Figure 5:**
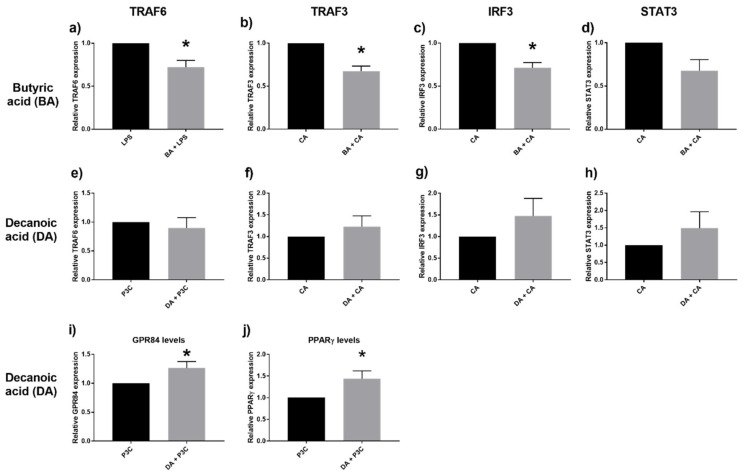
Relative expression of the genes TRAF6, TRAF3, IRF3, and STAT3 after stimulation with butyric acid (BA) 20 μM and LPS (**a**), CA (**b**–**d**), compared to decanoic acid (DA) 20 μM and P3C (**e**), CA (**f**–**h**). Gene expression levels of GPR84 (**i**) and PPARγ (**j**) following stimulation with decanoic acid 20 μM and P3C. Values are presented as mean ± SEM, results from at least 7 subjects were used. * *p* < 0.05 is the gene expression difference compared to the respective control (LPS/CA/P3C) groups (black bar, normalized to 1), Wilcoxon signed rank test was used to determine significance.

The presence of butyric acid led to reduced transcription of TRAF6. Similarly, TRAF3 and IRF3 transcription were also reduced (Figure 5a–c). However, decanoic acid did not affect TRAF6, TRAF3 and IRF3 expressions (Figure 5e–g). Given the effect of the respective fatty acids on IL-6 and IL-1β levels, we also looked at STAT3 (signal transducer and activator of transcription 3) expression. STAT3 transcription was not significantly influenced by either MCFA or SCFA (Figure 5d,h). GPR84 is a putative receptor for MCFA, and PPARγ is a receptor involved in lipid storage and glucose metabolism; both would be targets of interest pertaining to MCFA. Here, we found that decanoic acid induced up-regulation of GPR84 and PPARγ expression levels (Figure 5i,j).

### 2.6. Decanoic Acid Diminished HIF-1α Transcription

**Figure 6:**
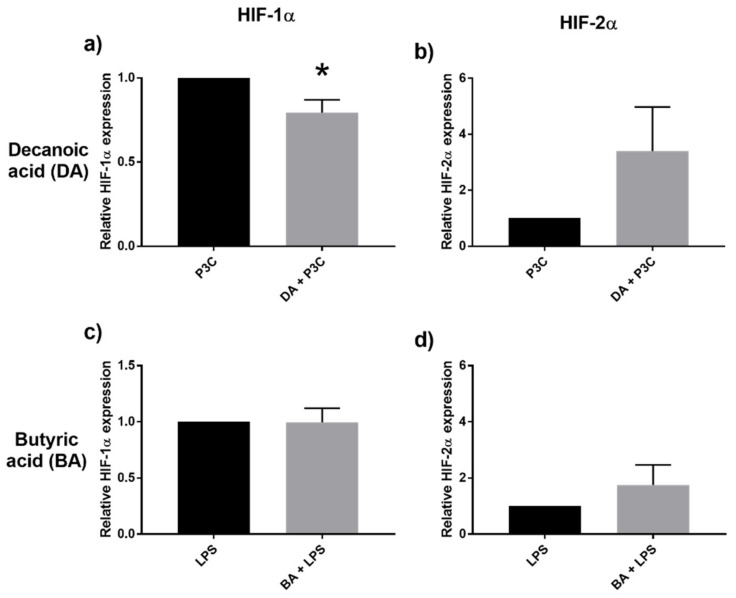
Relative expression of HIF1α (**a**), HIF2α (**b**) after stimulation with decanoic acid (DA) 20μM and P3C, and after stimulation with butyric acid (BA) 20 μM and LPS (**c**,**d**), assessed by qPCR. Values are presented as mean ± SEM, results from at least 7 subjects were used. * *p* < 0.05 is the gene expression difference compared to the respective control (P3C/LPS) groups (black bar, normalized to 1), Wilcoxon signed rank test was used to determine significance.

Hypoxia-inducible factor (HIF) proteins play an important role in cellular stress response and may be involved in fatty acid oxidation [21]. Of interest would be the effect exerted by MCFA on HIF-1α and HIF-2α. Notably, decanoic acid induced downregulation of HIF-1α with tendency to augment HIF-2α transcription (Figure 6a,b). Butyric acid, on the other hand, did not significantly change the HIF-1α/HIF-2α ratio (Figure 6c,d). 

## 3. Discussion

We have demonstrated that SCFA and MCFA exert divergent effects on immunity. SCFA attenuated host inflammatory response with reduction in IL-1β, IL-6, and TNFα. This was mediated predominantly through the TLR4 pathway and most evident with 4-carbon butyric acid, in the presence of LPS. In contrast, MCFA, best represented by 10-carbon decanoic acid, had the propensity to augment inflammation, not just with enhanced pro-inflammatory cytokine production, but resulting also in markedly diminished IL-10, through TLR2. 

The above findings inevitably raise postulations on why there should be such divergence on the influence on host immunity, by the range of fatty acids which differ over a range of carbon atoms. Looking further to the relative origin of these fatty acids, however, the major source of SCFA is intrinsic, attributable to dietary fibers as substrate for bacteria fermentation by intestinal *Firmicutes* and *Bacteroidetes*, resulting in SCFA production to the likes of butyrate and propionate [22]. Having said this, fermented food products, including cheese and yoghurt, also contain some SCFA [23]. On the other hand, the origin of MCFA is predominantly extrinsic from diet, of which rich sources include coconut oil and palm oil [5]. There is a basis of a physiological role for butyrate and other SCFA as metabolites in maintaining intestinal homeostasis. SCFA are recognized to play a role in maintaining intestinal epithelial integrity [4], which is to mitigate any inflammatory milieu at the epithelial mucosal interface. Indeed, inflammatory bowel disease is one well-described condition whereby intestinal butyrate levels are known to be low [24,25]. 

The selective basis of SCFA and MCFA for the TLR4 and TLR2 pathways, respectively is intriguing. A possible hypothesis is if TLR4, as a homodimer and signaling through MyD88/MAL and TRIF/TRAM, is evolutionarily conserved [26] as a receptor with sentinel role in regulating essential bodily function. The physiological production of SCFA de novo within the host intestine, whereby its plethoric roles include maintaining the mucosal epithelium and regulating cellular proliferation, is in line with this ascribed conserved signaling receptor characteristic of TLR4. The inhibition of inflammation induced by butyric acid as seen here through TLR4 and involving suppression of TRAF6 expression has been observed [27,28], On the other hand, TLR2 existing in the form of hetero-dimers as TLR2-TLR1, TLR2-TLR6 is thought to exhibit plasticity evolutionarily in its capacity to respond to the varied and changing environmental ligands [29] and pathogens including microbe- or damage-associated molecular patterns (MAMPs or DAMPs). With this perspective, MCFA, being primarily of extrinsic dietary origin, may putatively be ascribed for the propensity towards TLR2 on this account. 

*Candida*, as a gut commensal, was included as a complex ligand to study interaction with SCFA and MCFA. *Candida* is known to signal through TLR2 and TLR4 [18]. The modulatory consequence by SCFA and MCFA would have been an anticipated composite of TLR2/4. However, we observed that the down-modulatory effect of butyric acid on the proinflammatory cytokines was less pronounced here. Meanwhile the IL-6 and IL-1β augmentation by MCFA continued to be observed with *Candida*. We surmised if the above variant response by Candida might be accounted in part by involvement of other pathways triggered by *Candida* such as Dectin-1-CARD9 [18]. While the anti-inflammatory propensity of SCFA has been described by others [13,30] we further showed associated down-regulation of TRAF3 and IRF3 expression by butyric acid on *C. albicans*. This points to the varied scale of modulation induced by SCFA downstream of immune receptors in contrast to MCFA [31].

In the case of MCFA, we observed enhanced transcription of GPR84 and PPARγ. Both are putative receptors for MCFA which are induced under inflammatory conditions [32,33], and in turn further augment production of IL-6 and IL-1β. While PPARγ is thought to be involved in mediating insulin sensitivity and adipogenesis, the physiological roles played by both receptors, in particular GPR84, remains to be fully understood. Another unanticipated consequence of the immune regulation seen with MCFA was altered HIF-1α/HIF-2α ratio. HIF-α subunits are recognized transcription factors which regulate critical cellular metabolic and angiogenic functions in response to hypoxia and stress. HIF-1α is classically linked to regulate genes of pathways affecting metabolic processes glycolysis and NADPH regeneration [34], as would be expected in response to hypoxic stress conditions. Notably in our setting, decanoic acid-induced inflammatory stress resulted in reduced HIF-1α transcription and resultant relative preferential induction of HIF-2α over HIF-1α. Butyrate exposure did not alter HIF-1α expression, notwithstanding prior-reported observation that butyrate had the propensity to stabilize HIF-1 even under low oxygen conditions as in the small intestine [35]. HIF-2α has been perceived to regulate a more diverse range of target genes than HIF-1α including inflammatory and lipid metabolic pathways [34,36]. Our finding may be in line with this understanding that this altered HIF-1α/HIF-2α upregulation is a consequence of compensatory host response to the accentuated inflammation triggered by MCFA. 

Structurally, SCFA and MCFA differs by several carbon atoms, but their evoked immunological sequelae can be considerably divergent as we have demonstrated. This is attributable in part to SCFA being primarily of endogenous origin, generated de novo and playing a central role in intestinal epithelial immune regulation and homeostasis whereas MCFA are exogenously derived from diet with a notable inflammatory propensity. This understanding is of pertinence to consider when deriving specific fatty acid moieties or engineered probiotics and dietary modification to complement therapeutics in inflammatory intestinal diseases.

## 4. Materials and Methods 

### 4.1. Stimuli and Reagents

Heat-killed *Candida albicans* (CA) used as stimuli was prepared by growing live *Candida albicans* strain SC5314 until the germinating conidia stage. The germinating conidia was then washed, enumerated, resuspended in PBS and heat-killed by boiling in PBS at 100 °C for 1 h. Aliquots were kept at −20 °C and used in all subsequent experiments at a final concentration of 1 million cells per well (96 well plate). TLR4 ligand *Escherichia coli* lipopolysaccharide (LPS; *E. coli* K12 strain) (InvivoGen, San Diego, CA, USA) and TLR2 ligand Pam3Cys-Ser-(Lys)4, also known as tripalmitoyl S-glycerol-Cys-Ser-(Lys)4 (P3C) (EMC Microcollections, Tübingen, Germany) was reconstituted in endotoxin-free water (Gibco, ThermoFisher Scientific, MA, USA) according to manufacturer’s instructions and used at a final concentration of 10 ng/mL and 10 µg/mL, respectively. All the SCFA and MCFA were purchased from Sigma-Aldrich, St. Louis, MO, USA. The various SCFA and MCFA were measured, dissolved, and diluted accordingly to the concentration needed in RPMI culture medium supplemented with 10µg/mL gentamicin, 10 mM L-glutamine, and 10 mM pyruvate at 37 °C. 

### 4.2. PBMC Isolation and Stimulation Assays

Venous blood was drawn into sodium heparin tubes (BD Vacutainer ^®^, Franklin Lakes, NJ, USA) from healthy volunteers after informed consent. PBMC were isolated by density centrifugation on Ficoll–Hypaque (Pharmacia Biotech, Uppsala, Sweden). Cells were washed twice in saline, counted, and resuspended in RPMI culture medium supplemented with 10 µg/mL gentamicin, 10 mM L-glutamine, and 10 mM pyruvate. 

The stimulation experiments were performed in duplicates for PBMC isolated from each volunteer. 0.5 million cells were added into each well of a flat bottomed 96-well plate (Greiner Bio-One, Kremsmünster, Austria). The stimuli (LPS, P3C, CA) and fatty acids were dissolved in RPMI and added accordingly. The cells were incubated without serum in the media for 24 h to assay for IL-1β, IL-6, TNFα, or 48 h to assay for IL-10. The culture supernatants were collected after the incubation period and stored at −20 °C for the measurement of cytokine levels by ELISA, and the cell pellet used for RNA isolation and qPCR analysis. 

### 4.3. Cytokine Analysis by ELISA

Cytokine levels in culture supernatants were determined by ELISA using commercial kits specific for human TNFα, IL-1β, IL-6, IL-10 (ThermoFisher Scientific, MA, USA). Protocol and standard curves were according to kit instructions. Detection limits are as follows: IL-1β (1.17 pg/mL), IL-6 (1.56 pg/mL), TNF-α (4 pg/mL), and IL-10 (2.34 pg/mL).

### 4.4. RNA Isolation and Quantitative Reverse-Transcription PCR (qPCR) Analysis

Human PBMC from the stimulation assays were centrifuged at 700 G and the cell pellet was used for RNA isolation. RNA extraction was performed using TRIzol reagent (Sigma, St. Louis, MO, USA) according to extraction instructions. The RNA pellet was reconstituted in RNAse-free water (Invitrogen, Waltham, MA, USA). Reverse transcription PCR was done to get complementary DNA from the RNA, using oligo-dT primers (0.01 µg/mL) and SuperScript™ II Reverse Transcriptase, all from Invitrogen. qPCR was performed using the ABI Prism 7000 Thermocycler and GoTaq qPCR Mastermix (Promega Corporation, Madison, WI, USA). The primer sequences used are found in Table 1. Quantification of the qPCR signals for each sample was performed by comparing the cycle threshold (Ct) values for the gene of interest with the Ct values for Beta-2-microglobulin (B2M) as a housekeeping gene. Mean relative messenger RNA expression was calculated using the comparative Ct method. Values are expressed as a ratio of fold increase to mRNA levels of the control cells.

### 4.5. Statistical Analysis

The results from at least seven healthy volunteers are presented as mean ± standard error of the mean (SEM). The results were analyzed using GraphPad Prism (Version 7, San Diego, CA, USA), and Wilcoxon signed rank test was used for paired comparisons with the control (LPS/P3C/CA). The level of significance was set at *p* < 0.05. 

### 4.6. Ethics Statement

Institutional review board approval (from the Domain Specific Review Boards, National Healthcare Group, Singapore) was obtained to perform the studies involving human cells from healthy volunteers. 

## Figures and Tables

**Table 1:** Primer sequences used for qPCR to determine gene expression levels.

Primer (Human)	Sequence (5′-3′)	References
B2M_F	ATG AGT ATG CCT GCC GTG TG	[37,38]
B2M_R	CCA AAT GCG GCA TCT TCA AAC	[37,38]
STAT3_F	CAG CAG CTT GAC ACA CGG TA	[37]
STAT3_R	AAA CAC CAA AGT GGC ATG TGA	[37]
TRAF3_F	TCT TGA GGA AAG ACC TGC GAG	[38]
TRAF3_R	GCG ATC ATC GGA ACC TGA CT	[38]
TRAF6_F	TTG CCA TGA AAA GAT GCA GAG G	[38]
TRAF6_R	AGC CTG GGC CAA CAT TCT C	[38]
IRF3_F	AGA GGC TCG TGA TGG TCA AG	[38]
IRF3_R	AGG TCC ACA GTA TTC TCC AGG	[38]
PPARγ_F	CACAAGAACAGATCCAGTGGTTGCAG	[39]
PPARγ_R	AATAATAAGGTGGAGATGCAGGCTCC	[39]
GPR84_F	TTCAGCCCTTCTCTGTGGACA	[40]
GPR84_R	TGCAGAAGGTGGCACCG	[40]
HIF1α_F	TGCTCATCAGTTGCCACTTC	[41]
HIF1α_R	TCCTCACACGCAAATAGCTG	[41]
HIF2A_F	GAAGCGACAGCTGGAGTATG	[42]
HIF2A_R	TGAGGTTCTTCATCCGTTTCC	[42]

## Data Availability

Not applicable.

## Supplementary Materials

The following are available online at https://www.mdpi.com/article/10.3390/ijms22126453/s1.

## Abbreviations

CA	*Candida albicans*
ELISA	Enzyme-Linked Immunosorbent Assay
HIF	Hypoxia-Inducible Factor
IL-1β	Interleukin–1 Beta
IL-6	Interleukin–6
LPS	Lipopolysaccharide
MCFA	Medium Chain Fatty Acid(s)
PBMC	Peripheral Blood Mononuclear Cells
P3C	Pam-3-Cys
qPCR	Quantitative Reverse-Transcription PCR
SCFA	Short Chain Fatty Acid(s)
STAT	Signal Transducer and Activator of Transcription
TNFα	Tumour Necrosis Factor Alpha
TRAF	Tumor necrosis factor receptor–associated factor
