# Supplementary material for: The Divergent Immunomodulatory Effects of Short Chain Fatty Acids and Medium Chain Fatty Acids
Source: Int J Mol Sci. 2021 Jun 16;22(12):6453. doi: 10.3390/ijms22126453 (PMC8234078; doi:10.3390/ijms22126453)
Supplement: Supplementary file 1 [file ijms-22-06453-s001.zip › ijms-1232887-supplementary.pdf]

Supplementary Figure S1:

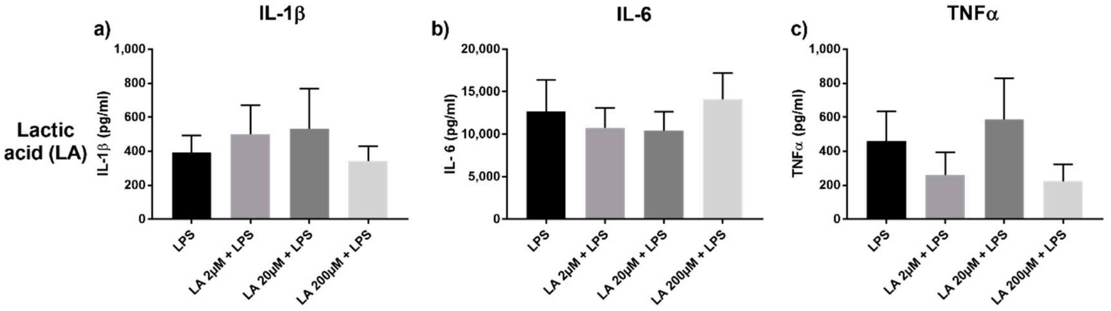

Supplementary Figure S1. Cytokine production of IL-1β, IL-6 and TNFα in the supernatants of PBMC stimulated with LPS and 2, 20, 200 μM of lactic acid, assessed by ELISA. Human PBMC were co-incubated with LPS and lactic acid (LA) (**a, b, c**). Values were presented as mean ± standard error of the mean (SEM), results from at least 7 subjects were used. No significance seen using Wilcoxon signed rank test for pair-wise comparisons with the LPS control (black bar).

Supplementary Figure S2:

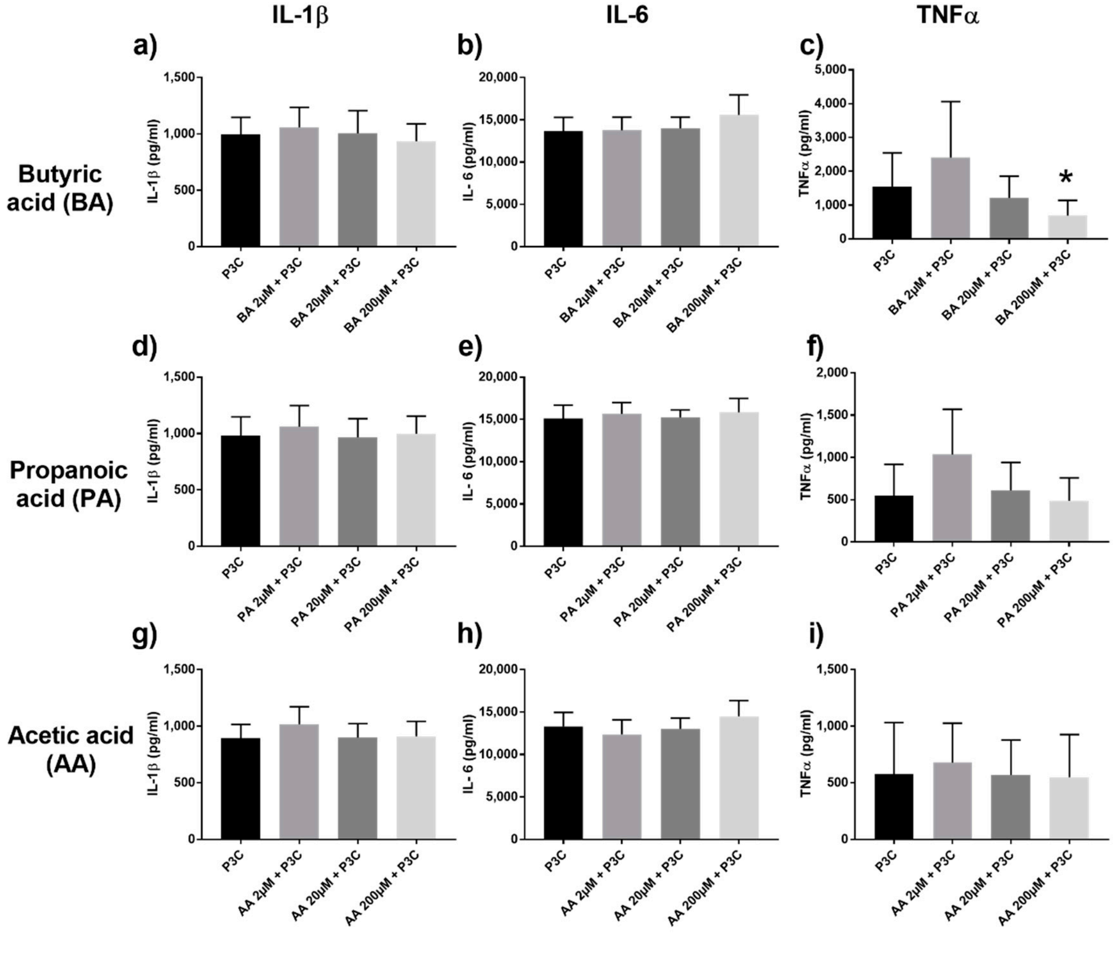

Supplementary Figure S2. Cytokine production of IL-1β, IL-6 and TNFα in the supernatants of PBMC stimulated with P3C and 2, 20, 200 μM of SCFA, assessed by ELISA. Human PBMC were co-incubated with P3C and butyric acid (BA) (**a, b, c**), propanoic acid (PA) (**d, e, f**), or acetic acid (AA) (**g, h, i**). Values

were presented as mean ± SEM, results from at least 7 subjects were used. Wilcoxon signed rank test to determine significance for pair-wise comparisons with the control, *p<0.05 is the cytokine level difference compared to the control P3C group (black bar).

Supplementary Figure S3:

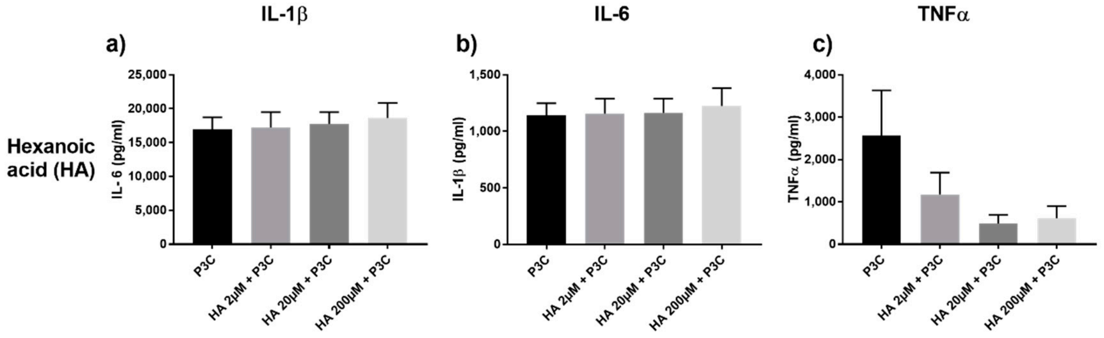

Supplementary Figure S3. Cytokine IL-1β, IL-6, and TNFα production in the supernatants of PBMC stimulated with P3C and 2, 20, 200 μM of hexanoic acid (HA) (**a, b, c**). Values were presented as mean ± SEM, results from at least 7 subjects were used. No significance seen using Wilcoxon signed rank test for pair-wise comparisons with the P3C control (black bar).

Supplementary Figure S4:

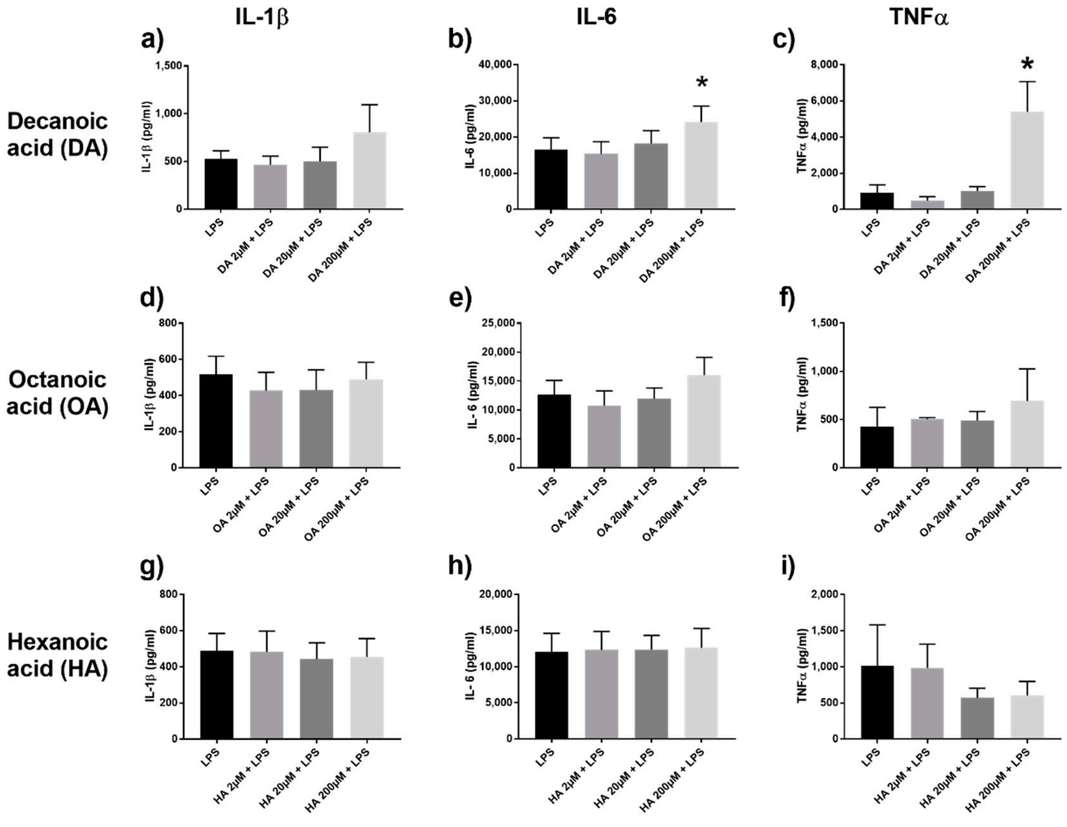

Supplementary Figure S4. Cytokine IL-1β, IL-6, and TNFα production in the supernatants of PBMC stimulated with LPS and 2, 20, 200 μM of decanoic acid (DA) (**a, b, c**), octanoic acid (OA) (**d, e, f**), hexanoic acid (HA) (**d, e, f**). Values were presented as mean ± SEM, results from at least 7 subjects were used. Wilcoxon signed rank test to determine significance for pair-wise comparisons with the control, *p<0.05 is the cytokine level difference compared to the control LPS group (black bar).

Supplementary Figure S5:

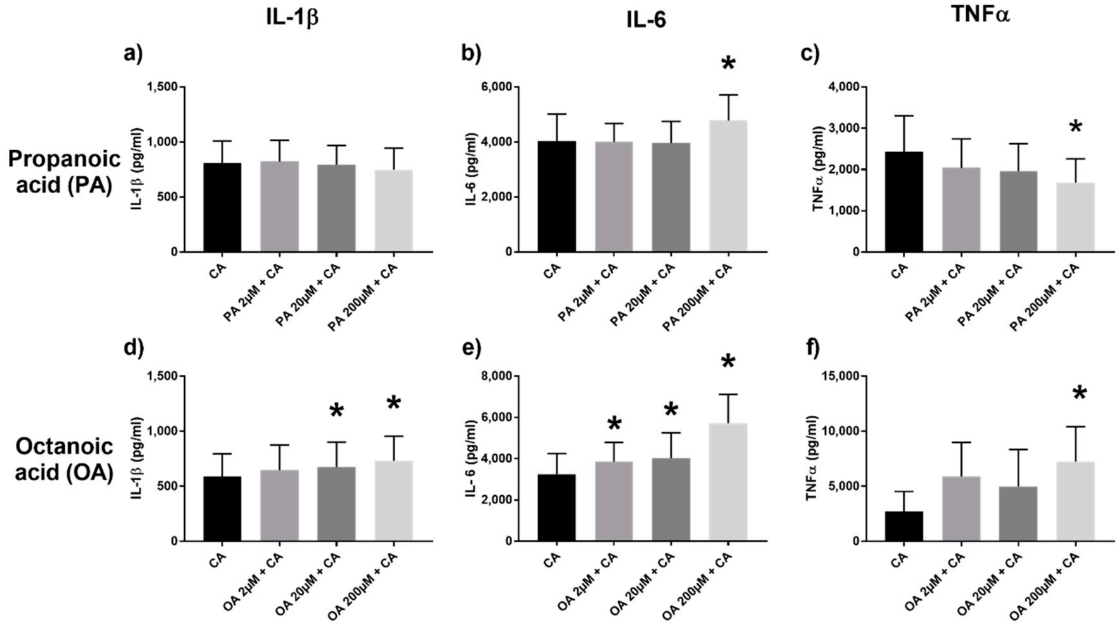

Supplementary Figure S5. Cytokine IL-1β, IL-6, and TNFα production in the supernatants of PBMC stimulated with CA and 2, 20, 200 μM of propanoic acid (PA) (**a, b, c**), and octanoic acid (OA) (**d, e, f**). Values were presented as mean ± SEM, results from at least 7 subjects were used. Wilcoxon signed rank test to determine significance for pair-wise comparisons with the control, *p<0.05 is the cytokine level difference compared to the control CA group (black bar).
